# Exposure prediction and dose optimization of polymyxin B based on bayesian and machine learning

**DOI:** 10.3389/fphar.2026.1859709

**Published:** 2026-06-26

**Authors:** Qihan Xu, Xuanyi Li, Shuqi Huang, Qin Ding, Yaqian Li, Nan Yang, Chenhui Deng, Bin Tang, Guoping Yang, Qi Pei

**Affiliations:** 1 Xiangya School of Pharmaceutical Sciences, Central South University, Changsha, China; 2 Department of Pharmacy, The Third Xiangya Hospital, Central South University, Changsha, China; 3 Linking Truth Technology Co., Ltd., Shanghai, China; 4 Changsha Kyan Pharmaceutical Technology Co., Ltd., Changsha, China; 5 Center of Clinical Pharmacology, The Third Xiangya Hospital, Central South University, Changsha, China; 6 National-Local Joint Engineering Laboratory of Drug Clinical Evaluation Technology, Changsha, China

**Keywords:** limited sampling strategy, maximum *a posteriori* Bayesian estimators, polymyxin B, population pharmacokinetics model, XGBoost

## Abstract

**Objectives:**

To explore the application scenarios of maximum *a posteriori* Bayesian estimation (MAP-BE) and eXtreme Gradient Boosting (XGBoost) in the prediction of polymyxin B (PMB) exposure.

**Methods:**

Two sets of simulations based on the population pharmacokinetic (PopPK) model developed for PMB were used for the development and testing of Bayesian and XGBoost models in four scenarios. The predictive performances of MAP-BE and XGBoost for the area under the concentration–time curve (AUC) over 12-h intervals at steady state were evaluated using root mean square error (RMSE), mean absolute error (MAE), and coefficient of determination (*R* ^2^).

**Results:**

Both MAP-BE and XGBoost accurately estimate the AUC_0–12h_ under a single sampling strategy using a dense point model (median [range] (mg·h/L): 3.32 [0.78–7.39] vs. 3.30 [0.95–7.04] for RMSE, 2.41 [0.54–5.53] vs. 2.61 [0.72–5.46] for MAE for MAP-BE and XGBoost, respectively). A single 6-h sampling strategy achieved the best prediction with negligible bias (RMSE<1 mg h/L, MAE<1 mg h/L, *R* ^2^ > 0.99). XGBoost was more accurate and efficient than MAP-BE for fitting single-trough concentrations. The performance of the 12-h XGBoost model allowed for temporal fluctuations in the 6-h range.

**Conclusion:**

This study provides evidence for the application scenario of MAP-BE and XGBoost for predicting the AUC of PMB, which assists in selecting better approaches when predicting drug exposure with available therapeutic drug monitoring information to guide the adjustment of dosing regimens in the clinic.

## Highlights

- Both MAP-BE and XGBoost have good predictions of AUC when dense concentrations of polymyxin B in patients are available for model development, and present the best prediction for concentrations at the sixth hour after administration.
- XGBoost has better prediction performance of AUC than MAP-BE when developing models with sparse concentrations, subject to consistent time points of modeling and prediction.
- The single-point XGBoost model has acceptable accuracy in predicting AUCs in the 6-h range.

## 1 Introduction

Due to the emergence of multi-resistant Gram-negative bacteria and the lack of new antibiotics, the lipopeptide antibiotic polymyxin B (PMB) has become the last line of defense against multi-drug-resistant Gram-negative bacteria (Huang et al., 2024; Yang et al., 2024). PMB does not require dose adjustment based on renal function and reaches mean steady-state blood concentrations more rapidly and with less pharmacokinetic (PK) variability than colistin methanesulfonate (CMS)/colistin. Therefore, PMB is a more appropriate choice for infections requiring rapid responses and maintenance of blood concentrations over a wide range of renal function (Yang et al., 2024; Fang et al., 2024).

The ratio of the area under the concentration–time curve (AUC) to minimum inhibitory concentrationis an important pharmacokinetics/pharmacodynamics index of PMB (Wang et al., 2023). The guidelines recommend a target of 50–100 mg h/L for the AUC at steady state over 24 h after PMB reaches a steady state, which corresponds to a mean steady-state plasma concentration of 2–4 mg/L (Tsuji et al., 2019). In practice, it is challenging to collect enough blood samples to obtain a full PK profile to calculate AUC using the unbiased trapezoidal method. Population pharmacokinetic (PopPK) models based on therapeutic drug monitoring (TDM) and accounting for patient variability are often used in combination with maximum *a posteriori* Bayesian estimation (MAP-BE) in clinical practice for AUC estimation. Studies have shown that the trough concentration (C_0_, pre-dose concentration), C_0_ combined with peak concentration (C_1_, at 1 h, end of infusion), C_0_ combined with C_2_ (at 2 h), and the four-point model (C_1_, C_1.5_, C_4_, and C_8_, where the subscript indicates hours after the start of infusion) are suitable for accurately estimating the AUC (Chen et al., 2021; Wang et al., 2020). However, a standard monitoring strategy for PMB has not yet been established. We are still unsure about the optimal time for TDM and how to use opportunity samples for precision dosing of PMB.

In recent years, the application of machine learning (ML) in the field of quantitative pharmacology has gained increasing attention. ML is able to efficiently and accurately process large data sets and identify algorithmic models that perform best with the lowest prediction errors (PEs) (Varela-Rey et al., 2024; Huang et al., 2025). Compared with quantitative pharmacological methods, data-driven ML is advantageous because of the amount of data it can process and its efficiency, but its output is less biologically interpretable (Keutzer et al., 2022). eXtreme Gradient Boosting (XGBoost) is a popular algorithm for ML. At each iteration, the greedy algorithm traverses all the features of each node from the root node to find the splitting point, the point with the highest score. After splitting to the maximum depth of the tree, it stops splitting and starts building the remaining part of the next tree; finally, it collects all the trees generated by the iterations to obtain the XGBoost model (Liu et al., 2021). XGBoost supports parallelization, fast training speeds, and various methods to prevent decision-tree overfitting, such as regularization model, shrinkage, and column subsampling (Wiens et al., 2025), which are superior to the traditional Gradient Boosting Decision Tree (GBDT) algorithm. Studies have reported use of the XGBoost algorithm for AUC estimation of drugs, and it has exhibited excellent predictive performance (Keutzer et al., 2022; Woillard et al., 2021a; Woillard et al., 2021b). The best way to develop such algorithms is to use real data (Bououda et al., 2022). However, it is difficult to obtain large amounts of real patient data in practice. The development of the XGBoost algorithm based on PopPK simulations may provide an alternative.

The purpose of this study was to compare the value of Bayesian methods and ML in predicting exposure to PMB and to explore the best sampling strategy for TDM to assist in individualized dosing in the clinic. The main goals of the study were (1) to develop a PopPK model based on routine TDM data after intravenous administration of PMB; (2) to train the XGBoost algorithm and to develop new PopPK models for MAP-BE based on a time–concentration series of PopPK model simulations; and (3) to evaluate the predictive performances of XGBoost and MAP-BE for PMB AUC_0–12h_ in four scenarios.

## 2 Materials and methods

**FIGURE 1:**
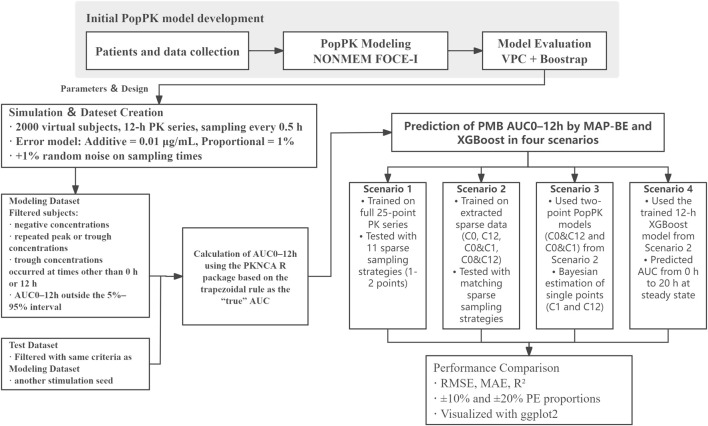
Study workflow for PMB AUC_0–12h_ prediction using PopPK modeling and XGBoost.

The overall study design and technical workflow are illustrated in Figure 1. Briefly, an initial PopPK model was developed for data simulation, followed by a performance comparison between MAP-BE and XGBoost in predicting PMB AUC_0–12h_ across four clinical scenarios.

### 2.1 Initial PopPK model development

#### 2.1.1 Patients and data collection

This study retrospectively included patients who had received intravenous PMB and at least one TDM in the intensive care unit of the Third Xiangya Hospital of Central South University from October 2019 to December 2020. The dosage was determined by the physician. Patient demographics (gender, age, height, and body weight), dosing regimen (time of administration, dose, and infusion rate), time of blood sample collection, sample concentration measurements, biochemical parameters, and receipt of continuous renal hemodialysis therapy were collected.

#### 2.1.2 Measurement of PMB concentrations

All plasma samples were bioassayed using ultra-performance liquid chromatography–tandem mass spectrometry with calibration ranges of 0.05–10 and 0.011–1.097 μg/mL for polymyxin B1 and B2, respectively. Full details are provided in a preprint (Zhang et al., 2026). This study was approved by the Ethics Committee of the Third Xiangya Hospital of Central South University.

#### 2.1.3 PopPK modeling

PopPK analysis was performed using nonlinear mixed effect modeling (NONMEM version 7.5) software assisted by Perl-Speaks-NONMEM (PsN, version 4.7.0; uupharmacometrics.github.io/PsN) and Pirana (version 2.9.2; www.pirana-software.com), and data visualization and statistical analysis were performed using the R package (Version 4.1.2; http://www.r-project.org). First-order conditional estimation with interaction was used for parameter estimation. One- or two-compartment structural models with first-order absorption, distribution, and elimination, and exponential interindividual variability were tested. Residual unknown variability was evaluated using proportional, additive, and mixed proportional–additive error models. The following covariates were tested using a two-stage, stepwise covariate modeling procedure (forward p-value<0.05; backward p-value<0.01): gender, age, height, body weight, white blood cells, hemoglobin, platelets, neutrophils, C-reactive protein, procalcitonin, alanine-transaminase, aspartate-transaminase, total bilirubin, direct bilirubin, albumin, globulin, total bile acids, blood urea nitrogen, serum creatinine, urea, creatinine clearance (CrCL), and receipt of continuous renal replacement therapy. Model selection during the development process was based on decreases in the objective function value (OFV) of 3.84, in shrinkage, and in the root square error of the parameter estimates and an improvement in the goodness-of-fit scatter plot.

#### 2.1.4 Evaluation of the final model

The final model was evaluated for predictive performance using a visual prediction check (VPC) with 1000 samples. In addition, a non-parametric bootstrap method with 1000 resamplings was performed to evaluate the robustness and precision of the parameter estimates.

### 2.2 Monte carlo simulations

#### 2.2.1 Simulation of modeling dataset and test dataset

To obtain more realistic PK profiles, the parameters of the developed PopPK model were used for simulations with the following modifications: additive error = 0.01 μg/mL proportional error = 0.01%. A PK series of 2000 virtual subjects over a 12-h interval at steady state was simulated using NONMEM software. The dosing regimen for simulation was set to 100 mg twice daily with an infusion rate of 100 mg/h, which is commonly used in our center (Table 2) and has also been adopted in several previous PMB PopPK and Monte Carlo simulation studies (Miglis et al., 2018; Wang et al., 2021).

Twenty-five sampling events (every 0.5 h) were set for each subject. One-percent random noise was added independently to the sampling time using the sdcMicro R package to achieve a more realistic clinical scenario. To ensure the reliability of the data, we excluded simulated subjects with the following characteristics: (1) subjects with negative concentrations; (2) subjects with repeated peak or trough concentrations; (3) subjects in which trough concentrations occurred at times other than 0 h or 12 h; and (4) subjects with AUC_0–12h_ outside the 5%–95% interval. After applying the above filtering procedure, the remaining subjects formed the modeling dataset for subsequent development of new PopPK models and training of XGBoost models.

An independent PK series containing 2000 virtual subjects was simulated with another seed number using NONMEM software. This series was filtered using the same criteria described above to form a test dataset for independent validation of the MAP-BE and XGBoost methods.

#### 2.2.2 Calculation of AUC_0–12h_

The AUCs were calculated over a 12-h interval for each subject in the simulated modeling and test datasets using the PKNCA R package based on the trapezoidal rule. The AUC_0–12h_ of the modeling dataset was used for training the XGBoost models, and the AUC_0–12h_ of the test dataset (AUC_ref_) was regarded as the “true” AUC.

### 2.3 Prediction of PMB AUC_0–12h_ by MAP-BE and XGBoost in four scenarios

The modeling and test datasets obtained from simulation, filtering, and AUC calculation in Section 2.2 were used to explore the AUC predictive performances of MAP-BE and XGBoost in four scenarios (Table 1).

**TABLE 1:** Four scenarios for modeling and AUC prediction.

Scenario	Theoretical time of modeling (h)	Theoretical time of prediction (h)	Method
1	Full concentration-time series	0, 0.5, 1, 2, 3, 4, 6, 8, 10, 12, 0 & 1	MAP-BE XGBoost
2	0, 12, 0 & 12, 0 & 1	0, 12, 0 & 12, 0 & 1	MAP-BE XGBoost
3	0 & 12, 0 & 1	1, 12	MAP-BE
4	12	0, 0.5, 1, 2, 3, 4, 5, 6, 7, 8, 9, 10, 11, 12, 13, 14, 15, 16, 17, 18, 19, 20	XGBoost

For XGBoost model training, simulated drug concentrations (e.g., peak concentration Cmax and trough concentration Cmin) were used as input features, with the AUC_0_–_12_h computed by the trapezoidal rule from the full simulated pharmacokinetic profiles serving as the label. The dataset was pre-split at the patient level into a training set (80%) and a test set (20%) to prevent data leakage. Hyperparameter tuning was performed using five-fold cross-validation on the training set, and the hyperparameter combination yielding the lowest average root mean square error (RMSE) was selected; the final model was subsequently retrained on the entire training set using this combination. Only the simulated concentration measurements were utilized as model inputs for AUC prediction, whereas patient covariates (e.g., body weight, age, or other clinical variables) were not included, as the objective was to directly learn the relationship between concentration data and AUC.

#### 2.3.1 Scenario 1: dense point PopPK and ML models for one- or two-point prediction

A two-compartment model with first-order elimination was developed using the full time–concentration series of the modeling dataset, and its final parameter estimates are provided in Supplementary Table 2. Individual PK parameters were assumed independent log-normally distributed:

$$\log\theta_{i,k}∼N\left(\log\theta_{\text{pop},k},\omega_{k}^{2}\right)$$

where the population estimates(θ_pop_) were: CL = 3.04 L/h, V1 = 40.6 L, V2 = 7.41 L, Q = 28.8 L/h. The between-subject variances (ω^2^) for CL and V1 were fixed to 0.161 and 0.319, respectively; V2 and Q were modeled without random effects. The residual error model was specified as:

$$C_{\text{obs},ij}∼N\left(C_{\text{pred},ij},\sigma_{ij}^{2}\right),\sigma_{ij}=\sqrt{0.01^{2}+{\left(0.01\cdot C_{\text{pred},ij}\right)}^{2}}$$

According to Bayes’ theorem, the posterior distribution of the individual PK parameters was defined as:

$$p\left(\theta_{i}|C_{i}\right)\propto p\left(C_{i}|\theta_{i}\right)p\left(\theta_{i}\right),{\hat{\theta }}_{i}={\mathrm{argmax}}_{\theta_{i}}\;p\left(\theta_{i}|C_{i}\right)$$

MAP-BE was conducted using this model with 11 sampling strategies (C_0_, C_0.5_, C_1_, C_2_, C_3_, C_4_, C_6_, C_8_, C_10_, C_12_, and C_0_ & C_1_;where C_x_ denotes the plasma concentration at x hours after the start of PMB infusion) derived from the test dataset. The predicted time–concentration series were used to calculate AUC_0–12h_.

Training and optimization of the ML models were performed using python (version 3.10) software. The XGBoost algorithm was trained on the full PK series of the modeling dataset as well as the AUCs calculated by the trapezoidal rule, with time and concentration as the features and AUC_0–12h_ as the label. To prevent overfitting of the model, a five-fold cross-validation was carried out to tune the hyperparameters and evaluate model performance. The XGBoost model was tested using 10 independent datasets derived from the test dataset, consistent with the sampling strategies described in 2.3.1 (C_0_, C_0.5_, C_1_, C_2_, C_3_, C_4_, C_6_, C_8_, C_10_, C_12_, and C_0_ & C_1_), to predict the AUC_0–12h_.

#### 2.3.2 Scenario 2: one- or two-point PopPK and ML models for corresponding time point predictions

Four subsets of the PK series with theoretical times of 0 h, 12 h, 0 h & 12 h, and 0 h & 1 h in the modeling dataset were extracted separately to develop the PopPK models with sparse data. These models were used for MAP-BE with the corresponding sampling strategies (C_0_, C_12_, C_0_ & C_12_, and C_0_ and C_1_) for the test dataset, respectively. The AUC_0–12h_s under different sampling strategies were calculated from the time–concentration series predicted by MAP-BE.

Similarly, the concentration data of 0 h, 12 h, 0 h, 12 h, 0 h and 1 h of the modeling dataset and the calculated AUC_0–12h_s were extracted for training the XGBoost algorithm. The algorithm was validated using the concentration data of the corresponding times in the test dataset to estimate AUC_0–12h_.

#### 2.3.3 Scenario 3: two-point PopPK model for single time point predictions

The 0 h and 12 h and 0 h and 1 h PopPK models described in 2.3.2 were used to perform Bayesian estimations of C_1_ and C_12_ of the test dataset to evaluate the performance of MAP-BE for the two-point sparse model for single point prediction.

#### 2.3.4 Scenario 4: single-point ML model for series time point predictions

The trained 12-h XGBoost model (described in 2.3.2) was used to perform AUC prediction from 0 h to 20 h after the last dose at steady state to assess the effect of time fluctuations on the predictive performance of the ML model.

### 2.4 Comparison of the performances of MAP-BE and ML in AUC prediction

The RMSE(mg·h/L) (Equation 1), mean absolute error (MAE, mg·h/L) (Equation 2), and coefficient of determination (*R* ^2^) (Equation 3) between the AUC_0–12h_s predicted by the PopPK and ML models developed using dense or sparse data and the AUC_ref_ were calculated to evaluate and compare the accuracy and precision of MAP-BE and ML in predicting AUC_0–12h_ under different scenarios. The proportion of simulated subjects with AUC_0–12h_ PEs outside 10% and 20% was also calculated. In addition, visualization of AUC_0–12h_ and the PE results was conducted using the ggplot2 R package.

$$RMSE=\sqrt{{\left(1/n\right)\sum_{i=1}^{n}\left({obs}_{i}‐{pred}_{i}\right)}^{2}}$$ (1)

$$MAE=\left(1/n\right)\sum_{i=1}^{n}\left|{obs}_{i}‐{pred}_{i}\right|$$ (2)

$$R^{2}=1‐\sum {\left({obs}_{i}‐{pred}_{i}\right)}^{2}/\sum {\left({obs}_{i}‐mean\left(obs\right)\right)}^{2}$$ (3)

where obs_i_ is the individual AUC_0–12h_ value of the simulated test dataset from the PopPK model, and pred_i_ is the individual AUC_0–12h_ value predicted by MAP-BE or XGBoost.

## 3 Results

### 3.1 Development and evaluation of the initial PopPK model

Ninety-two plasma samples from 20 patients (17 males and three females) were used for the development of the PopPK model of PMB. The patient characteristics are summarized in Table 2 and the distribution of plasma samples per patient across time intervals is provided in Supplementary Figure 1. A two-compartment model with first-order elimination best described the data. The model included interindividual variability (IIV) on clearance (CL) and distribution volume of the central compartment (V1) and explained the residual variability with a mixed additive–proportional error model. No covariates were included in the final model in the two-stage stepwise covariate modeling procedure. The parameter estimates of the final model and the bootstrap results are summarized in Table 3. The normalized prediction distribution errors (NPDE) from 1000 Monte Carlo simulations had a mean of 0.0485 (SE = 0.11) and a variance of 1.08 (SE = 0.16), close to the theoretical N (0,1) distribution, while the global test was non-significant (p = 0.346). The NPDE diagnostic plot showed no significant errors or biases of the model (Supplementary Figure 2). The goodness-of-fit test and VPC showed no significant errors or biases of the model (Supplementary Figures 3, 4). These results further support the adequacy and robustness of the final model.

**TABLE 2:** Patients characteristics.

Characteristic	
Number of patients	20
Demographics	
Gender (male), n(%)	17 (85%)
Age (years), median (range)	53.5 (27–90)
Body weight (kg), median (range)	64 (40–120)
Height (cm), median (range)	169 (155–175)
Dosing regimen	
Dose (mg, twice daily), median (range)	100 (50–200)
infusion Rate (mg/h), median (range)	100 (33.33–200)
Biochemical parameters	
Alanine-transaminase (IU/L), median (range)	25.5 (6–648)
Aspartate-transaminase (IU/L), median (range)	50 (10–2534)
Total bilirubin (μmol/L), median (range)	29 (2.11–186.40)
Total bile acid (μmol/L), median (range)	11.5 (2.20–137.50)
Albumin (g/L), median (range)	28.2 (19.10–61.00)
Serum creatinine (mg/dL), median (range)	2.06 (0.17–4.65)
Creatinine clearance rate (mL/min), median (range)	47.38 (20.07–422.44)
Blood urea nitrogen, median (range)	17.6 (5.9–53.9)
Uric acid (μmol/L), median (range)	201.5 (6.5–675.0)
C-reactive protein (mg/L), median (range)	149.1 (15.2–306.0)
Procalcitonin (ng/mL), median (range)	3.1 (0.3–68.1)
White blood cell count(×109/L), median (range)	10.5 (0.8–22.2)
Hemoglobin (g/L), median (range)	76.5 (7.4–111.0)
Platelets (×109/L), median (range)	130.5 (4.7–517.0)
Neutrophils (%), median (range)	86.7 (64.0–95.6)
Clinical characteristics	
Continuous renal replacement therapy, n(%)	7 (35%)

**TABLE 3:** Population pharmacokinetics parameters of final model.

Parameter	Final model		Bootstrap	
	Estimate	RSE (%)	Median	95% CI
Fixed effects				
CL (L/h)	3.05	11	3.10	2.41–4.25
V1 (L)	40.3	11	41.93	30.33–82.17
V2 (L)	7.52	9	6.91	2.34–17.14
Q (L/h)	29.7	14	27.41	15.96–40.49
Random effects				
IIV on CL (%)	29.1	35	29.02	12.43–70.84
IIV on V1 (%)	33.3	44	34.18	7.11–115.4
Residual variability				
Proportional error (%)	19.2	46	17.97	4.10–25.83
Additive error (mg/L)	0.06	7	0.06	0.02–0.29

Abbreviations: CL, clearance; V1, distribution volume of the central compartment; V2, distribution volume of the peripheral compartment; Q, intercompartmental clearance; IIV, inter-individual variability; RSE, relative standard error; CI, confidence interval.

### 3.2 Monte carlo simulations

After applying the aforementioned filters, the final available modeling dataset contained 39,475 concentrations from 1,579 subjects (three subjects contained negative concentration values, one subject had repeated trough concentrations, 241 subjects had trough concentrations at times other than 0 h or 12 h, and 176 subjects had AUC_0–12h_s outside the 5%–95% interval). The final available test dataset contained 39,250 concentrations from 1,570 subjects (five subjects contained negative concentration values, two subjects contained repeated peak and trough concentrations, 247 subjects had trough concentrations at times other than 0 h or 12 h, and 176 subjects had AUC_0–12h_s outside the 5%–95% interval). The distribution of the final retained modeling and testing dataset is summarized in Supplementary Table 1.

### 3.3 Prediction of PMB AUC_0–12h_ by MAP-BE and XGBoost in four scenarios

#### 3.3.1 Scenario 1: dense point PopPK and ML models for one- or two-point predictions

In the PopPK analysis, the full time–concentration series of the modeling dataset was best described by a two-compartment model with first-order elimination (Supplementary Table 2). The ML model was successfully trained for the full time-concentration-AUC series using the XGBoost algorithm with tuned hyperparameters (Supplementary Table 3).

**FIGURE 2:**
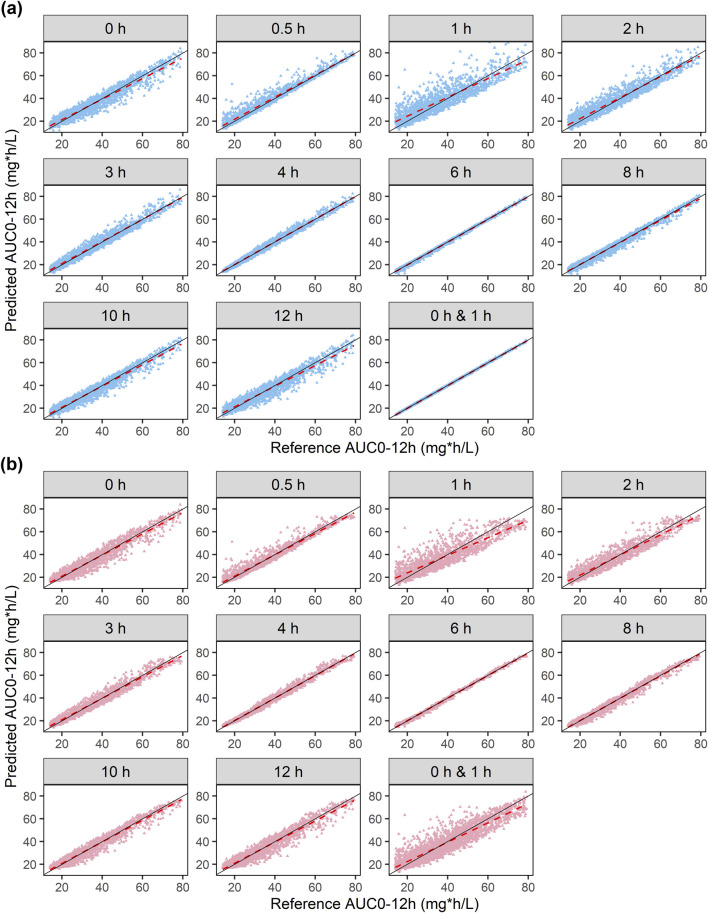
Scatter plots showing the correlation between predicted AUC_0–12h_ and the trapezoidal rule reference values in Scenario 1. **(a)** represents the MAP-BE method, and **(b)** represents the XGBoost model. Each subplot corresponds to different sampling time points or combinations. The solid black line indicates the line of identity (y = x), and the dashed red line represents the regression fit.

**FIGURE 3:**
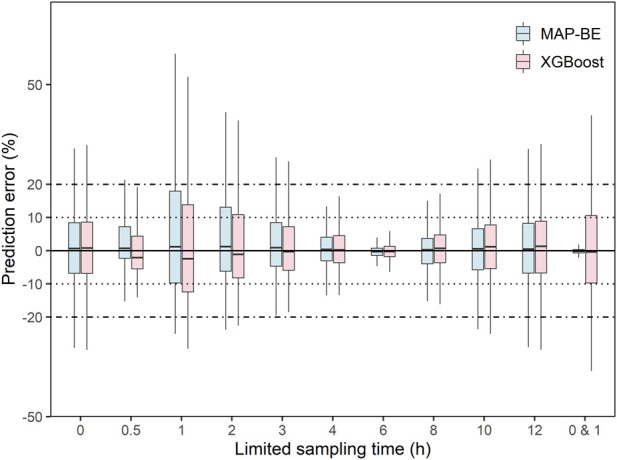
Boxplot of prediction error of AUC_0–12h_ predicted by MAP-BE or XGBoost in Scenario 1.

AUC predictions under different limited sampling strategies were performed using the PopPK and ML models developed with dense data. The median (range) of the AUC_0–12h_ estimated by MAP-BE and XGBoost is summarized in Supplementary Table 4. The MAE, RMSE, and *R* ^2^ values of AUC_0–12h_s predicted by MAP-BE and XGBoost for 11 sampling strategies when modeling using simulated rich data and the percentage of subjects with PEs outside ±10% and ±20% are integrated in Table 4. When predicting AUC_0–12h_ at a single sampling time, both MAP-BE and XGBoost exhibited desirable predictive performance (median [range]: 3.32 [0.78–7.39] mg·h/L vs. 3.30 [0.95–7.04] mg·h/L for RMSE, 2.41 [0.54–5.53] mg·h/L vs. 2.61 [0.72–5.46] mg·h/L for MAE, and 0.954 [0.777–0.998] vs. 0.952 [0.784–0.996] for *R* ^2^). Except for 1 h, all single sampling strategies achieved MAEs and RMSEs less than 5 mg h/L, R^2^s greater than 0.90, and proportions of subjects with PEs outside of ±10% and ±20% of less than 50% and 20%, respectively. The distribution and PEs between the predicted and reference AUC_0–12h_s were visualized with a scatter plot (Figure 2) and box plot (Figure 3), respectively. The upper quartile (Q1) and lower quartile (Q3) of the PE of the AUC_0–12h_ for all examined sampling strategies were within ±20%. Sampling strategy C_6_ resulted in the best predictive performances using either MAP-BE (RMSE = 0.78 mg h/L, MAE = 0.54 mg h/L, and *R* ^2^ = 0.998) or XGBoost (RMSE = 0.95 mg h/L, MAE = 0.72 mg h/L, and *R* ^2^ = 0.996), with small biases and high accuracies. When using two sampling points (C_0_ & C_1_) for prediction, MAP-BE outperformed the predictions by all single sampling strategies (RMSE = 0.28 mg h/L, MAE = 0.21 mg h/L, and *R* ^2^ = 1.00). However, the predictive precision of XGBoost did not significantly improve compared with that of predictions with single sampling strategies (RMSE = 5.76 mg h/L, MAE = 4.32 mg h/L, and *R* ^2^ = 0.855).

**TABLE 4:** Performance comparison of MAP-BE and XGBoost for estimating AUC_0–12h_ when modeling with dense data.

Theoretical time of tested limited sampling (h)	RMSE (mg·h/L)		MAE (mg·h/L)		*R* ^2^		PE out of ±10%, n (%)		PE out of ±20%, n (%)	
	MAP-BE	XGBoost	MAP-BE	XGBoost	MAP-BE	XGBoost	MAP-BE	XGBoost	MAP-BE	XGBoost
0	4.12	4.09	3.17	3.19	0.927	0.927	38.79	40.19	12.17	11.15
0.5	3.48	3.27	2.17	2.33	0.954	0.953	20.51	18.85	9.36	6.75
1	7.39	7.04	5.53	5.46	0.777	0.784	59.87	61.40	26.94	25.61
2	4.64	4.52	3.60	3.62	0.911	0.912	43.50	45.16	15.54	13.69
3	3.06	3.04	2.41	2.46	0.961	0.960	27.32	27.39	5.48	5.22
4	1.76	1.82	1.38	1.46	0.987	0.985	3.82	6.75	0.00	0.00
6	0.78	0.95	0.54	0.72	0.998	0.996	1.97	3.63	0.25	0.06
8	2.16	2.21	1.64	1.73	0.980	0.979	15.80	14.65	1.53	1.40
10	3.32	3.30	2.57	2.61	0.952	0.952	31.46	32.80	7.07	6.37
12	4.05	4.06	3.13	3.16	0.929	0.928	39.30	40.06	11.91	11.21
0 & 1	0.28	5.76	0.21	4.32	1.000	0.855	0.00	50.80	0.00	18.38

#### 3.3.2 Scenario 2: one- or two-point PopPK and ML models for corresponding time point predictions

Four one-compartment PopPK models were developed using PK information for 0 h, 12 h, 0 h, 12 h, 0 h, and 1 h in the modeling dataset, and their parameter estimates are shown in Supplementary Table 5. The hyperparameter tuning results for the 4 ML models trained with these PK series are summarized in Supplementary Table 6. The median (range) of the AUC_0–12h_ estimated by MAP-BE and XGBoost is summarized in Supplementary Table 7.

**FIGURE 4:**
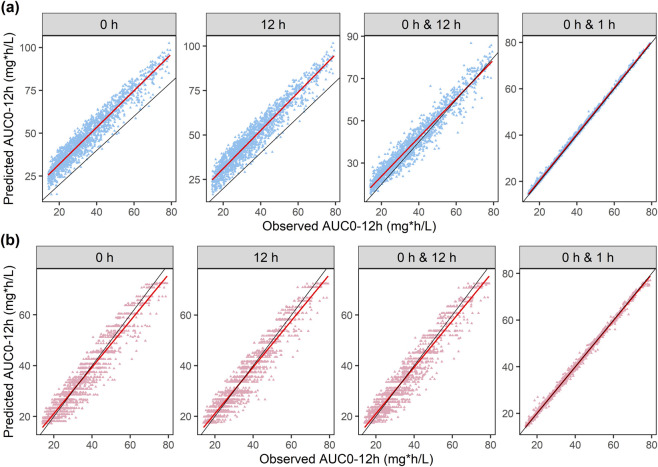
Scatter plots of AUC0-12h predicted by MAP-BE **(a)** and XGBoost **(b)** in Scenario 2 *versus* the reference AUC_0–12h_ calculated by the trapezoidal rule.

**FIGURE 5:**
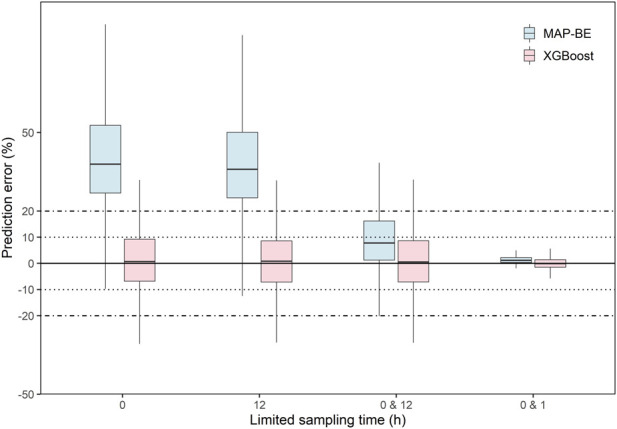
Boxplot of prediction error of AUC_0–12h_ predicted by MAP-BE or XGBoost in Scenario 2.

The MAE, RMSE, *R* ^2^ values of AUC_0–12h_ predicted by MAP-BE and XGBoost in scenario 2 and the percentage of subjects with PEs outside ±10% and ±20% are integrated in Table 5. The scatter plot of AUC (Figure 4) and the box plot of PE (Figure 5) were used to visualize the prediction results. The single valley concentration (C_0_ or C_12_) PopPK model lacks sufficient robustness, as evidenced by its poor predictive performance of MAP-BE (RMSE>10 mg h/L, MAE>10 mg h/L, and percentage of predicton error (PE) outside ±10% > 90%). The performance of MAP-BE was significantly improved when models with two trough concentrations (C_0_ and C_12_) were used (RMSE<5 mg h/L, MAE<5 mg h/L, and percentage of PE outside ±10% < 50%). In contrast, XGBoost showed good and similar predictions when models with either a single trough concentration (C_0_ or C_12_) or two trough concentrations (C_0_ and C_12_) were used. In addition, when modeling with trough and peak concentrations (C_0_ and C_1_), both MAP-BE and XGBoost predicted AUCs under the corresponding sampling strategies with high accuracies and small biases (RMSE<1 mg h/L, MAE<1 mg h/L, *R* ^2^ > 0.99, and percentage of PE outside ±10% < 1%).

**TABLE 5:** Performance comparison of MAP-BE and XGBoost for estimating AUC_0–12h_ when modeling with sparse data.

Theoretical time of tested limited sampling (h)	MSE (h·mg/L)		MAE (h·mg/L)		*R* ^2^		PE out of ±10%, n (%)		PE out of ±20%, n (%)	
	MAP-BE	XGBoost	MAP-BE	XGBoost	MAP-BE	XGBoost	MAP-BE	XGBoost	MAP-BE	XGBoost
0	14.00	4.14	13.16	3.21	0.923	0.923	96.94	41.15	96.94	12.10
12	13.16	4.14	12.30	3.21	0.925	0.926	96.18	40.00	96.18	11.78
0 and 12	4.48	4.12	3.65	3.21	0.938	0.926	47.01	40.25	47.01	11.53
0 and 1	0.68	0.90	0.51	0.64	0.999	0.996	0.51	0.83	0.51	0.13

Abbreviations: RMSE, root mean square error; MAE, mean absolute error; PE, prediction error.

#### 3.3.3 Scenario 3: two-point PopPK model for single time point predictions

**FIGURE 6:**
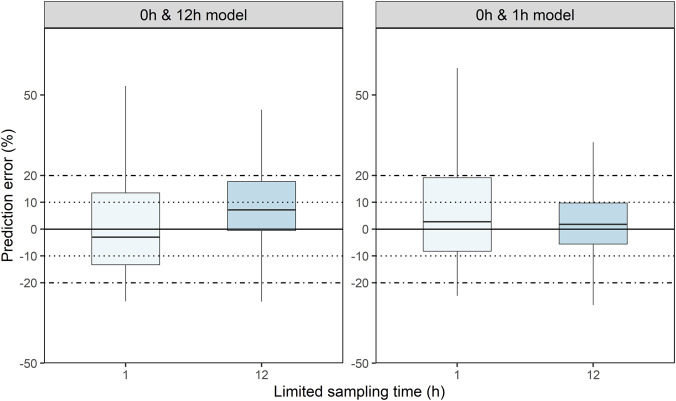
Boxplot of AUC_0–12h_ for C1 and C12 predicted by MAP-BE using 0 h and 12 h model or 0 h and 1 h model.

MAP-BE was performed using the PopPK model developed with C_0_, C_12_, C_0_, and C_1_ for 1-h or 12-h PK series of the test dataset, and the PEs are presented as box plots in Figure 6. All PEs were acceptable (PE of Q1 and Q3 were within 20%), which implies that the two-point Bayesian model can estimate the AUC_0–12h_ of a single point well.

#### 3.3.4 Scenario 4: single-point ML model for series time point predictions

**FIGURE 7:**
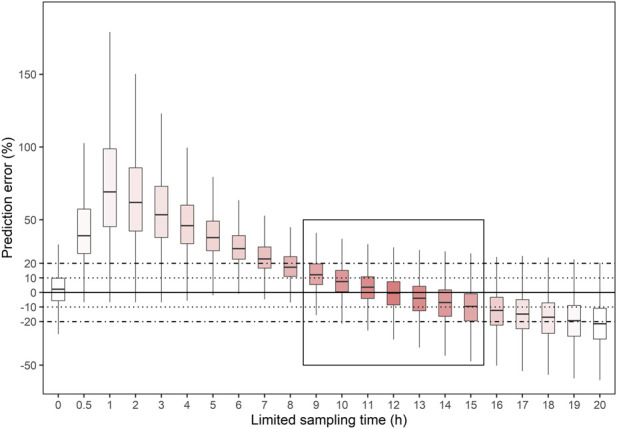
Impact of sampling time on prediction accuracy. The boxplots illustrate the distribution of percentage prediction errors for AUC_0–12h_ using the XGBoost model trained with a 12 h reference window. A rectangular box highlights the optimal sampling window from 9 h to 15 h, characterized by minimal bias and high precision, with the majority of predictions falling within the ±20% threshold.

The PEs of the 12-h XGBoost model predicting the AUC_0–12h_ over a series of individual sampling time points is shown in Figure 7. As the time point of limited sampling gradually moved away from 12 h, the PE increased. Nevertheless, the 12-h XGBoost model produced acceptable estimates of the AUC_0–12h_ for both the pre- and post 3-h (9–15 h) periods (PEs of Q1 and Q3 were within 20%).

### 3.4 Applications

An online program (https://pmb.xy3yx.com/) was developed to predict the AUC of PMB using the XGBoost dense point model (Scenario 1) and the 0&1h model (Scenario 2) and to obtain dose recommendations based on the criteria of AUC_ss, 0–24h_ in the range of 50–100 mg h/L.

## 4 Discussion

To explore the performances of the PopPK model-based Bayesian approach and the ML model in predicting PMB exposure, we developed a PopPK model of PMB exposure and performed two sets of simulations for model development and testing of these two approaches. PopPK and ML models developed using simulated dense (full time–concentration series) and sparse (0 h, 12 h, 0 h, 12 h, 0 h, and 1 h) data were used to investigate the predictive performance of MAP-BE and XGBoost for PMB AUC_0–12h_ in four scenarios. We found that a single sampling point, C_6_, provided the most accurate AUC_0–12h_ estimate. Comparison of the AUC_0–12h_s predicted by MAP-BE and XGBoost with the AUC_ref_ indicated that both methods have acceptable accuracy and precision for all sampling strategies tested in the model developed using dense concentrations. However, the two approaches performed differently in the sparse data model: XGBoost was better at estimation, and MAP-BE was not robust. This study determined the applicable scenarios for MAP-BE and XGBoost estimation of PMB AUC_0–12h_, provided evidence for the utility of multiple PMB monitoring strategies, and confirmed the value of PopPK in combination with MAP-BE and ML for precision dosing.

In clinical practice, steady-state AUC is closely associated with the efficacy and toxicity of PMB, and CL is the primary determinant of AUC. In this study, a two-compartment model of PMB was developed based on TDM concentrations used in routine care, and the PK parameters estimated were generally comparable with previously published PopPK models of PMB.The estimated CL in our model was 3.05 L/h, which was slightly higher than the previously reported values of 2.5 L/h (Manchandani et al., 2018) and 2.63 L/h (Miglis et al., 2018), indicating generally comparable clearance estimates across studies.

Clinical variables including CrCL were not retained in the final model. In particular, the influence of CrCL on PMB clearance remains controversial across previous PopPK studies. The U.S. Food and Drug Administration-approved drug label on PMB recommends dose reduction for patients with renal impairment (Polymyxin B For Injection, 2022). However, CrCL correlates poorly with CL because PMB is predominantly excreted *via* non-renal routes, and the standard dose of PMB exposure is similar in patients with differing renal functions (Zavascki et al., 2008; Chen et al., 2022; Yu et al., 2025). Therefore, dose adjustment of PMB based on renal function is not required. Previous PopPK studies have reported inconsistent findings regarding the influence of CrCL on PMB clearance. While some studies identified CrCL as a significant covariate (Manchandani et al., 2018; Hanafin et al., 2023), others did not retain it in their final models (Miglis et al., 2018; Sandri et al., 2013; Kubin et al., 2018). Notably, both positive and negative findings have been reported in studies with relatively small and larger sample sizes, suggesting that the discrepant results cannot be explained solely by sample size. Nevertheless, the relatively limited sample size of the present study may have reduced the power to detect subtle covariate effects. The interpretation of PK variability of PMB by CrCL must be validated in a larger population.

In Scenario 1 of dense data modeling (Figures 2, 3), XGBoost and MAP-BE performed similarly when predicting AUC_0–12h_ using single concentrations. However, XGBoost does not perform better than MAP-BE using the sampling strategy of C_0_ combined with C_1_. This may be because the XGBoost algorithm cannot identify the changes in the two concentrations over time in the same subject; this prevented the combined sampling strategy from improving the model’s predictive performance. Therefore, the XGBoost algorithm requires a consistent input of limited sampling times during model training and testing to maximize its predictive performance. This supports the conclusion of Woillard et al. (2021a): when using the XGBoost algorithm to predict the AUC of mycophenolic acid, the XGBoost model trained on three concentrations could not manage more than three samples per patient.

C_6_ was the best single sampling strategy for both the both MAP-BE and XGBoost methods, which was consistent with the literature (Chen et al., 2021). Sample information close to the peak concentration, such as C_1_ and C_2_, resulted in relatively poor predictions. This may be because deviations in sampling times have a greater effect on the pharmacokinetics near the highest point of the time–concentration curve. Any deviation from the peak concentration will result in a decrease in concentration, leading to an underestimation of the true AUC_0–12h_ (Bououda et al., 2022).

In Scenario 2 (Figures 4, 5), we examined the performances of the one- and two-point sparse data models for AUC_0–12h_ estimation at the corresponding times using the trough or peak concentrations commonly monitored in clinical practice. We found it was difficult to achieve a satisfactory fit with PopPK models for single trough concentrations, even though they were described by one-compartment models. When the *a priori* concentration was increased to two, the PE of MAP-BE was markedly reduced, especially for the 0 h and 1 h model, and achieved excellent accuracy and precision. In contrast, data from sparsely sampled points can be well trained for ML models if sufficient samples are available. In addition, the development of models for sparse data made the efficiency of XGBoost even more significant. The PopPK models were developed in a step-by-step, manually-tuned and repeated process (Keutzer et al., 2022), and a single run can take minutes to hours. In contrast, ML typically runs once, and it takes only a few seconds to train the model. Compared with the data-driven algorithm of ML, PopPK models can better describe the physiological mechanisms of drug absorption, distribution, metabolism, and elimination (Keutzer et al., 2022; Woillard et al., 2021b). However, for the prediction of AUC_0–12h_ in sparse data scenarios, we believe the best model selected by the XGBoost algorithm with minimal PE can be a complementary tool to MAP-BE.

The XGBoost algorithm has excellent running speed and predictive accuracy but is less flexible than the Bayesian approach. Scenario 3 (Figure 6) revealed that the two-point Bayesian model (0 h, 12 h, 0 h, and 1 h) exhibits acceptable performance in predicting the AUC_0–12h_ of a single point (1 h and 12 h). However, it was difficult to apply ML to this scenario due to the limitations of the input format. In addition, data-driven ML algorithms need homogeneous training and test data to obtain satisfactory predictive performance, which is reflected in Scenario 4 (Figure 7). During the elimination of the drug, the predictive ability of the 12-h model for individual points becomes progressively worse as time moves away from 12 h, with acceptable performance demonstrated in the 6-h range (9–15 h). However, the model also provided good predictions for 0 h, which may be due to the similar PK characteristics of the trough concentrations C_0_ and C_12_.

We acknowledge the limitations of this study. Some potential covariates may not have been included in the final model due to the limited patient data, resulting in inadequate explanations of the variability in PK parameters. Due to the lack of sufficient realistic time–concentration series, we performed MAP-BE and XGBoost predictions based on data simulated by the PopPK model. Although this model has performed well in internal evaluations, it has not been subjected to more rigorous external evaluations, which makes it difficult to use our new models with patient populations not included in the initial model. Furthermore, XGBoost was chosen as the ML algorithm in this study. Given the type of dataset and application scenario in this study, where AUC was predicted under a limited sampling strategy using only drug concentration measurements as inputs, XGBoost may be the optimal algorithm for this task, as demonstrated in previous studies (Woillard et al., 2021a; De Lucca Thomaz et al., 2026; Woillard et al., 2021c). Nevertheless, future studies incorporating multiple ML algorithms are warranted to further evaluate model robustness and confirm the optimal approach for PMB AUC prediction. Studies using real-world data to evaluate the performance of MAP-BE and ML to predict AUC_0–12h_ should be conducted.

## 5 Conclusion

This study compared the performances of MAP-BE and XGBoost in predicting the AUC_0–12h_ of PMB in four scenarios based on two PopPK simulation data sets. We showed that the AUC_0–12h_ for one or two or more time points can be estimated well using Bayesian estimation when dense data were available, while an increase in time points could not improve the performance of the ML model with dense data. C_6_ provided the best estimate of a single sampling strategy in the dense data model. In sparse data models, robust and efficient ML models should be used if only one concentration is available. Sparse data models with two or more time points should be subjected to Bayesian or ML methods, depending on the practicalities of modeling and application. In conclusion, ML is valuable in conjunction with PopPK and Bayesian estimation for precision dosing in clinical practice.

## Data Availability

The original contributions presented in the study are included in the article/Supplementary Material, further inquiries can be directed to the corresponding author.
