# Supplementary material for: Exposure prediction and dose optimization of polymyxin B based on bayesian and machine learning
Source: Front Pharmacol. 2026 Jun 26;17:1859709. doi: 10.3389/fphar.2026.1859709 (PMC13349818; doi:10.3389/fphar.2026.1859709)
Supplement: Supplementary file 1 [file DataSheet1.PDF]

Exposure Prediction and Dose Optimization of Polymyxin B Based on Bayesian and Machine Learning

Qihan Xu [1,†], Xuanyi Li [1,†], Shuqi Huang [2], Qin Ding[2], Yaqian Li [2], Nan Yang [2], Chenhui Deng [3], Bin Tang [4], Guoping Yang [1, 2, 5, 6], Qi Pei [1, 2, *]

[1] Xiangya School of Pharmaceutical Sciences, Central South University, Changsha, China;

[2] Department of Pharmacy, The Third Xiangya Hospital, Central South University, Changsha, China;

[3] Linking Truth Technology co., Ltd., Shanghai, China;

[4] Changsha Kyan Pharmaceutical Technology co., Ltd., Changsha, China;

[5] Center of Clinical Pharmacology, The Third Xiangya Hospital, Central South University, Changsha, China;

[6] National-Local Joint Engineering Laboratory of Drug Clinical Evaluation Technology, Changsha, China;

[†]These authors contributed equally to this work and share first authorship

*** Corresponding author**

Qi Pei, Department of Pharmacy, The Third Xiangya Hospital, Central South University, 172 Tongzipo Road, Changsha 410013, China. Tel.: +86 731 8861 8338. Fax: +86 731 8861 8338. Email: peiqi1028@126.com.

Supplementary table 1 Characteristic of simulated modeling and testing datasets

Theoretical time (h)	Modeling dataset (n=1579)		Test dataset (n=1570)	
	**Time deviation, median (range)**	**Concentration (mg/L), median (range)**	**Time deviation, median (range)**	**Concentration (mg/L), median (range)**
0	0.00 (-0.12-0.11)	1.86 (0.14-5.78)	0.00 (-0.11-0.11)	1.90 (0.06-6.21)
0.5	0.00 (-0.10-0.12)	3.02 (1.25-7.21)	0.00 (-0.10-0.12)	3.04 (1.25-6.97)
1	0.00 (-0.10-0.12)	4.01 (1.59-9.27)	0.00 (-0.10-0.12)	4.07 (1.51-9.24)
2	0.00 (-0.14-0.13)	3.62 (1.50-8.20)	0.00 (-0.14-0.11)	3.63 (1.46-8.02)
3	0.00 (-0.13-0.13)	3.37 (1.41-7.58)	0.00 (-0.13-0.13)	3.38 (1.43-7.84)
4	0.00 (-0.13-0.11)	3.12 (1.33-7.27)	0.00 (-0.11-0.11)	3.17 (1.39-7.31)
6	0.00 (-0.14-0.10)	2.71 (0.91-6.52)	0.00 (-0.14-0.10)	2.77 (0.72-6.67)
8	0.00 (-0.12-0.12)	2.38 (0.47-6.15)	0.00 (-0.10-0.12)	2.39 (0.33-6.47)
10	0.00 (-0.11-0.11)	2.09 (0.26-5.98)	0.00 (-0.11-0.11)	2.08 (0.14-6.26)
12	0.00 (-0.11-0.11)	1.86 (0.15-5.79)	0.00 (-0.11-0.11)	1.87 (0.17-6.23)

Supplementary table 2 New PopPK model developed with rich data

Parameter	Estimate	RSE (%)
Fixed effects		
CL (L/h)	3.04	1
V1 (L)	40.6	1
V2 (L)	7.41	2
Q (L/h)	28.8	4
Random effects		
IIV on CL (%)	16.1	3
IIV on V1 (%)	31.9	4
Residual variability		
Proportional error (%)	1	1
Additive error (mg/L)	0.01	5

Abbreviations： CL, clearance; V1, distribution volume of the central compartment; V2, distribution volume of the peripheral compartment; Q, intercompartmental clearance; IIV, inter-individual variability; RSE, relative standard error; CI, confidence interval.

Supplementary table 3 Best tuned parameter values of model trained with rich data

Parameter	Value
learning rate	0.1
n_estimators	190
max_depth	3
min_child_weight	7
gamma	0
subsample	1
colsample_bytree	1

Supplementary table 4 Estimation of AUC_{0-12h} by MAP-BE and XGBoost when modeling with dense data

Theoretical time of tested limited sampling (h)	AUC_{0-12h} estimated by MAP-BE, median (range)	AUC_{0-12h} estimated by XGBoost, median (range)
0	32.53 (11.81-83.99)	32.57 (16.36-83.83)
0.5	34.56 (13.49-81.31)	33.01 (15.54-74.94)
1	35.17 (12.51-94.06)	33.35 (13.10-71.84)
2	33.88 (12.49-85.02)	32.65 (13.51-73.85)
3	33.38 (12.82-85.88)	32.69 (14.11-75.97)
4	33.40 (13.48-82.46)	32.87 (15.23-77.61)
6	33.02 (11.83-79.22)	33.08 (14.57-76.70)
8	32.44 (11.84-80.86)	32.71 (14.93-76.45)
10	32.26 (11.87-81.57)	32.99 (16.00-76.13)
12	32.46 (12.65-84.17)	32.51 (16.57-78.02)
0 & 1	33.32 (13.87-79.53)	33.12 (13.10-83.83)

Supplementary table 5 New PopPK models developed with sparse data

Parameter	0h		12h		0h & 12h		0h & 1h	
	Estimate	RSE (%)	Estimate	RSE (%)	Estimate	RSE (%)	Estimate	RSE (%)
Fixed effects								
CL (L/h)	2.16	6	2.2	6	2.84	1	3.00	1
Vd (L)	17.7	13	18.5	14	38.8	2	47.1	1
Random effects								
IIV on CL (%)	10.8	7	11.1	7	13.7	3	15.8	3
IIV on Vd (%)	0 fixed	-	0 fixed	-	23.9	8	22.6	4
Residual variability								
Proportional error (%)	0.32 fixed	-	0.32 fixed	-	1	10	0.32 fixed	-
Additive error (mg/L)	0.003	20	0.004	7	0.013	22	0.072	64

Abbreviations: CL, clearance; Vd, distribution volume; IIV, inter-individual variability; RSE, relative standard error.

Supplementary table 6 Best tuned parameter values of model trained with sparse data

Parameter	Value			
	0h	12h	0h & 12h	0h & 1h
learning rate	0.1	0.1	0.1	0.1
n_estimators	50	50	50	190
max_depth	2	2	2	3
min_child_weight	1	2	3	5
gamma	0	0	0	0.2
subsample	0.6	0.8	0.8	0.9
colsample_bytree	0.6	0.6	1	1

Supplementary table 7 Estimation of AUC_{0-12h} by MAP-BE and XGBoost when modeling with sparse data

Theoretical time of tested limited sampling (h)	AUC_{0-12h} estimated by MAP-BE, median (range)	AUC_{0-12h} estimated by XGBoost, median (range)
0	46.16 (14.21-102.60)	33.32 (17.23-72.35)
12	45.40 (16.54-101.66)	33.55 (17.42-72.41)
0 & 12	35.32 (15.11-86.77)	33.53 (17.35-72.43)
0 & 1	34.00 (14.37-79.69)	33.37 (13.97-77.02)

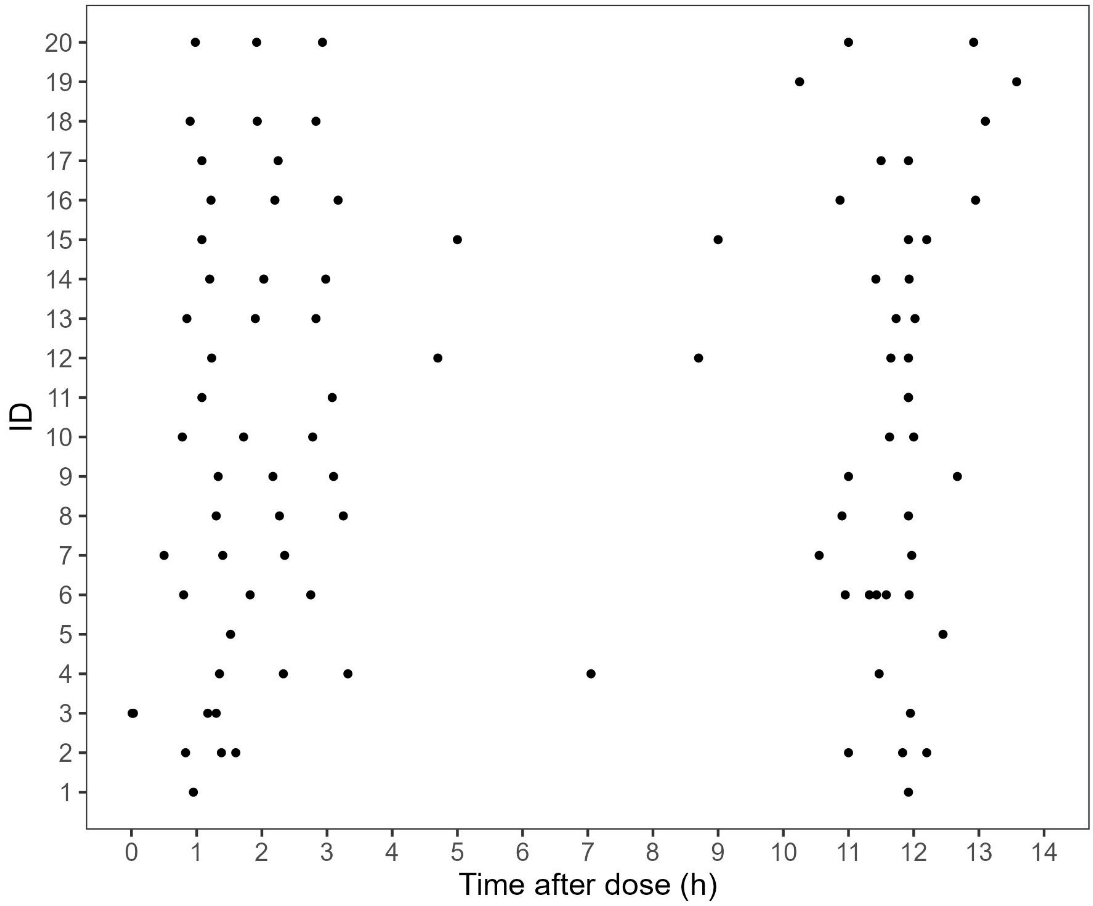

Supplementary Figure 1. Scatter plot of observed concentrations versus time after dose (TAD) stratified by patient.

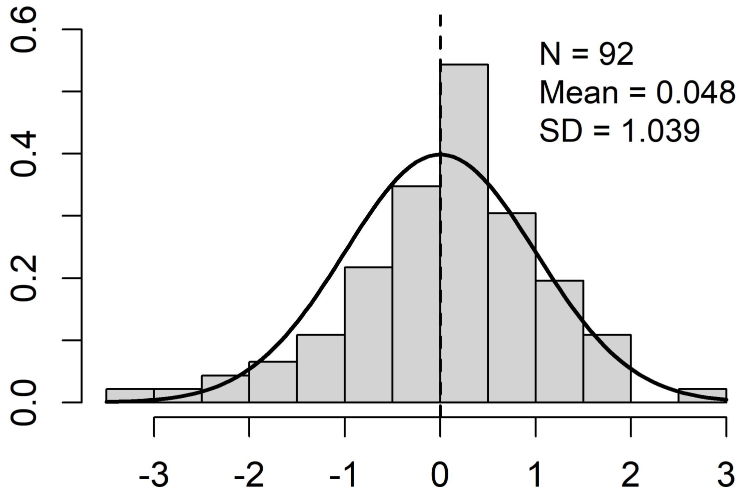

Supplementary Figure 2. Distribution of normalized prediction distribution errors (NPDE).

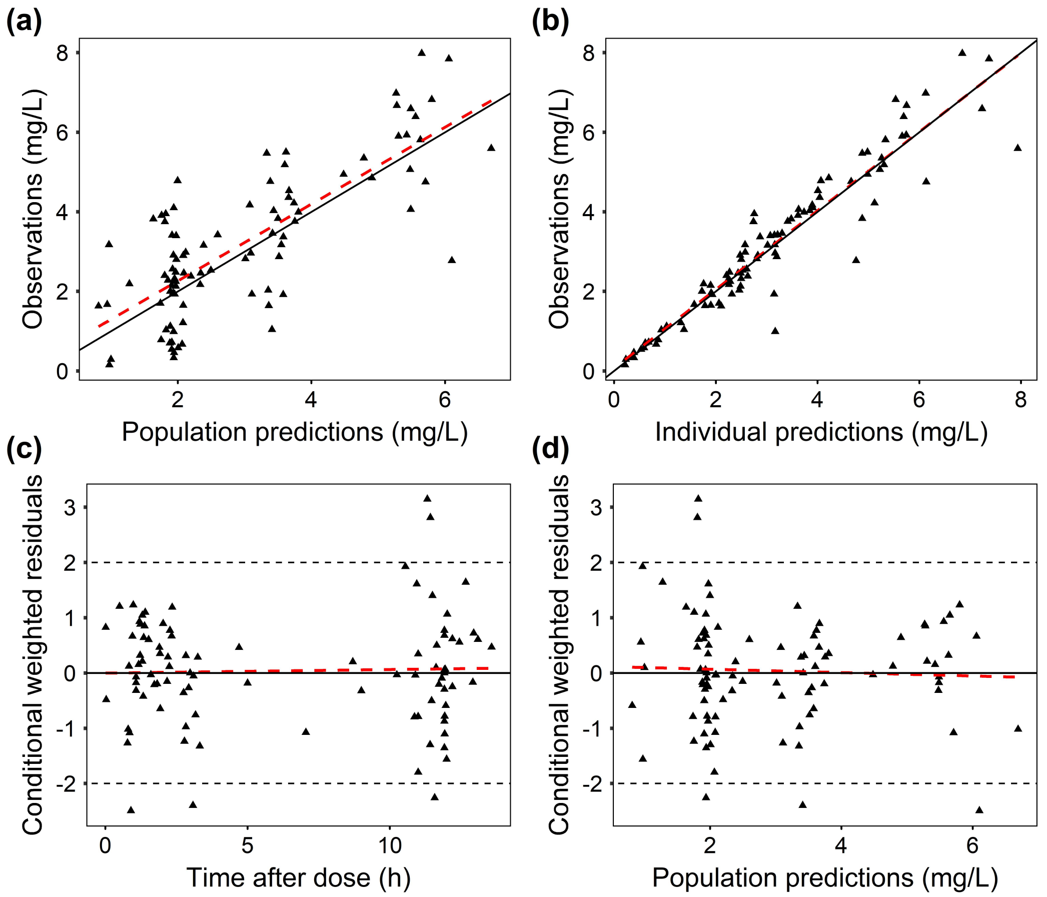

Supplementary Figure 3 Goodness-of-fit plots of the final model: **(a)** observations versus population predictions; **(b)** observations versus individual predictions; **(c)** conditional weighted residuals versus time after dose; **(d)** conditional weighted residuals versus population predictions.

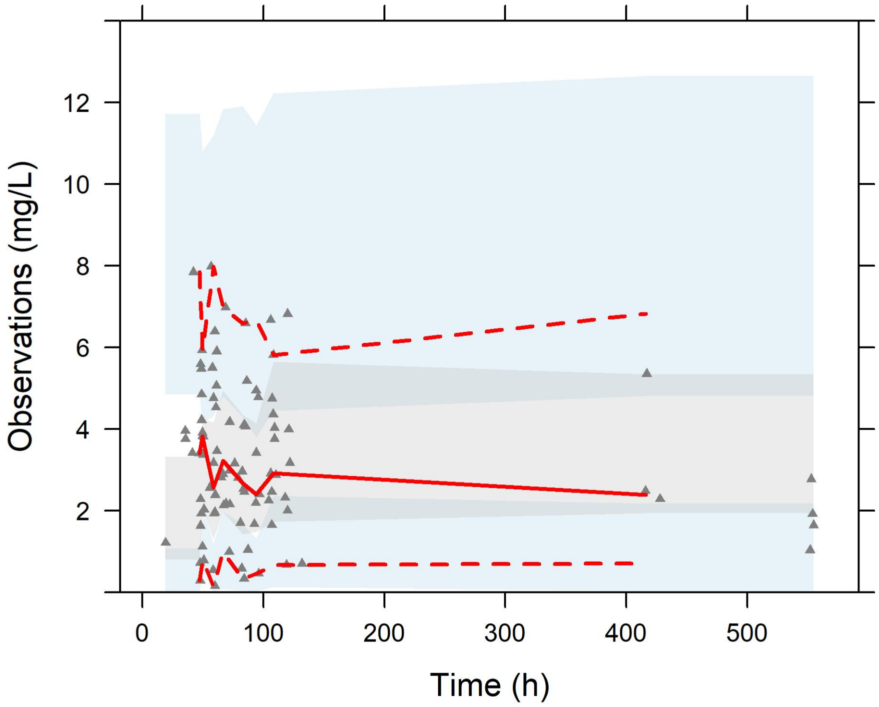

Supplementary Figure 4 Visual predictive check (VPC) of the final model. Solid triangles represent the observations, and red lines represent the 5th, 50th and 95th percentiles of the observed data. The purple-shaded areas represent the 95% confidence intervals around the simulated 5th, 50th, and 95th percentiles.
